# Hemodynamic Signal Changes During Motor Imagery Task Performance Are Associated With the Degree of Motor Task Learning

**DOI:** 10.3389/fnhum.2021.603069

**Published:** 2021-04-15

**Authors:** Naoki Iso, Takefumi Moriuchi, Kengo Fujiwara, Moemi Matsuo, Wataru Mitsunaga, Takashi Hasegawa, Fumiko Iso, Kilchoon Cho, Makoto Suzuki, Toshio Higashi

**Affiliations:** ^1^Faculty of Health Sciences, Tokyo Kasei University, Saitama, Japan; ^2^Department of Occupational Therapy, Nagasaki University Graduate School of Biomedical Sciences and Health Sciences, Nagasaki, Japan; ^3^Department of Health Sciences, Nagasaki University Graduate School of Biomedical Sciences, Nagasaki, Japan

**Keywords:** motor imagery, motor learning, oxygenated hemoglobin, ball rotation task, motor area

## Abstract

**Purpose:**

This study aimed to investigate whether oxygenated hemoglobin (oxy-Hb) generated during a motor imagery (MI) task is associated with the motor learning level of the task.

**Methods:**

We included 16 right-handed healthy participants who were trained to perform a ball rotation (BR) task. Hemodynamic brain activity was measured using near-infrared spectroscopy to monitor changes in oxy-Hb concentration during the BR MI task. The experimental protocol used a block design, and measurements were performed three times before and after the initial training of the BR task as well as after the final training. The BR count during training was also measured. Furthermore, subjective vividness of MI was evaluated three times after NIRS measurement using the Visual Analog Scale (VAS).

**Results:**

The results showed that the number of BRs increased significantly with training (*P* < 0.001). VAS scores also improved with training (*P* < 0.001). Furthermore, oxy-Hb concentration and the region of interest (ROI) showed a main effect (*P* = 0.001). An interaction was confirmed (*P* < 0.001), and it was ascertained that the change in oxy-Hb concentrations due to training was different for each ROI. The most significant predictor of subjective MI vividness was supplementary motor area (SMA) oxy-Hb concentration (coefficient = 0.365).

**Discussion:**

Hemodynamic brain activity during MI tasks may be correlated with task motor learning levels, since significant changes in oxy-Hb concentrations were observed following initial and final training in the SMA. In particular, hemodynamic brain activity in the SMA was suggested to reflect the MI vividness of participants.

## Introduction

To effectively perform motor imagery (MI), it is important to ensure the vividness of participants’ MI in objective terms. MI is defined as mental rehearsal in which an individual simulates an objective action within the brain without performing actual motions, and similar brain activation caused by performing motions is observed (Jeannerod, 2001; Kimberley et al., 2006). MI allows rehearsal without performing the actual motions and has been used as a tool for practicing in the sports field (Guillot and Collet, 2008; MacIntyre et al., 2018). Performing MI repeatedly, which is called mental practice, has been used not only in the sports field, but also in the field of rehabilitation in recent years. MI can also be executed in patients who have difficulty performing motions, particularly in those with cerebrovascular disease, and it has been reported as an effective method for improving motor function (Page et al., 2001, 2011; Liu et al., 2004; Sharma et al., 2006; Riccio et al., 2010). As a treatment strategy, MI is regarded as a method for complementing motion performances due to the exhibition of similar brain activation to that caused by performing motions, which has been reported to change brain plasticity (Ruffino et al., 2017; Li et al., 2018; Yoxon and Welsh, 2019).

It is important to evaluate the effectiveness of MI to induce similar brain activation to that caused by performing motions. Participants’ MI ability, clarity, means, and experience in MI tasks are factors that have been shown to affect effectiveness (Mulder et al., 2004; Mulder, 2007; Schuster et al., 2011). To assess the MI ability, questionnaires such as the Kinesthetic and Visual Imagery Questionnaire (KVIQ) (Malouin et al., 2007) and the Revised Movement Imagery Questionnaire (Gregg et al., 2010) have been developed and used in the clinical setting. The Visual Analog Scale (VAS) has been used for the assessment of the subjective clarity of MI, and it has an advantage in that the clarity can be assessed for each task of MI used in mental practice (Lotze and Halsband, 2006; Ikeda et al., 2012). However, these assessments of MI ability and MI clarity are subjective and the clarity with which the participants can perform MI cannot be objectively assessed.

Therefore, we consider that neurophysiological assessment is necessary for directing brain activation during MI similar to that during motion. There have been reports of studies on brain activity during MI using brain imaging devices such as functional magnetic resonance imaging (fMRI) and positron emission tomography (PET) (Stephan et al., 1995; Ruby and Decety, 2003; Solodkin et al., 2004; Hanakawa et al., 2008). Studies using fMRI and PET showed deactivation of the primary motor cortex, and activation of the premotor cortex (PMA) and the supplementary motor area (SMA) (Hanakawa et al., 2003; Lotze et al., 2003; Cunnington et al., 2005). Previous neurophysiological studies using transcranial magnetic stimulation (TMS) have also reported excitatory changes in the primary motor cortex during MI (Kasai et al., 1997; Stinear and Byblow, 2003; Pelgrims et al., 2011). The results regarding the activation sites in the brain vary depending on the device. In addition, some devices are highly restrictive; thus, it is not easy to use such devices for assessment in actual rehabilitation settings. Although spatial resolution is inferior to PET and fMRI, near-infrared spectroscopy (NIRS) can monitor brain activation in the region of interest by devising probe placement. Therefore, we have been studying whether NIRS can be used as a neurophysiological assessment method. NIRS is highly portable and widely applicable, which allows its use during bedside care and treatment since it is less restrictive and non-invasive. Thus, it could be easily used in the field of rehabilitation. Several studies have examined brain activation during MI using NIRS and cerebral hemodynamics during MI of a tapping task (Iso et al., 2016), swallowing (Kober and Wood, 2014), and eating have been shown (Matsuo et al., 2020). In addition, several studies have aimed to increase the effect of MI by feeding the cerebral hemodynamics measured by NIRS back to patients to enhance brain activation (Mihara et al., 2012; Kober et al., 2014, 2018; Ota et al., 2020).

However, although the experience in tasks and the degree of learning have been shown to affect MI (Mulder et al., 2004; Mulder, 2007; Schuster et al., 2011), no studies have examined how much they affect the changes in cerebral hemodynamics during MI. We previously examined the performance of motions and cerebral hemodynamics during MI (Iso et al., 2016), and the effects of the dominant/non-dominant hand (Matsuo et al., 2020). The results showed an increase in cerebral hemodynamic change in the PMA and SMA, comparable to that observed during exercise. While TMS or fMRI studies have already reported the effects of experience and competence in MI tasks on the excitability of the primary motor cortex (Szameitat et al., 2007; Tsukazaki et al., 2012; Wriessnegger et al., 2014), no NIRS studies have determined such effects. Moreover, there are no NIRS studies that have examined the association between task competence and activity in the SMA involved in motor learning. To develop future neurophysiological assessment methods using NIRS, it is necessary to determine the cerebral hemodynamics of each region associated with MI by considering the effects of the experience in tasks and the degree of learning.

The objective of the present study was to examine the relationship between changes in cerebral hemodynamics during MI and the degree of task learning. The degree of task learning was examined using a ball rotation (BR) task, which has been used in many studies (Nojima et al., 2012; Suzuki et al., 2013; Horiba et al., 2019). We examined motor-related areas that exhibit the equivalent level of activation to that during the performance of motions using NIRS.

## Materials and Methods

### Participants

The target sample size of this study was based on 80% statistical power to detect changes in task learning with a 0.40 effect size and a two-sided α-level of 0.05. A sample size of 10 was calculated by G^∗^Power (Faul et al., 2007, 2009). The participants were 16 neurologically healthy right-handed adults (age: 31.6 ± 4.0, male: 13, female: 3). Hand dominance was determined using the Edinburgh Handedness Inventory (Oldfield, 1971). None of the participants had switched handedness. The MI abilities of the participants were evaluated using the KVIQ (77.8 ± 2.24 points). The present study was approved by the ethics committee of the medical corporation Toujinkai and conducted after we obtained consent for participation in writing from all the participants.

### Experimental Procedure

**FIGURE 1:**
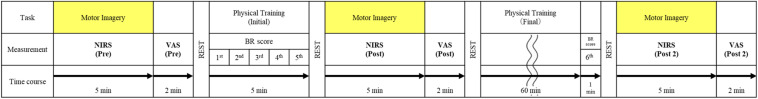
The experimental protocol. The physical practice (initial and final training) was performed in three phases classified as pre, post, and post 2. The training was performed in six sessions. The black arrow shows the timeline of this protocol. The horizontal row of task represents the MI of ball rotation or physical practice in each item. The initial training consisted of five sessions of 1 min each. The final training consisted of 60 min of self-training and 1 min of one session. MI, motor imagery; BR, ball rotation task; NIRS, near-infrared spectroscopy; VAS, visual analog scale.

The participants sat on a comfortable chair and placed their hands on the table. The experimental task was a BR in the palm, in which the learning of the motion can be expected to be achieved by short-term practice of the actual motion (Kawashima et al., 1998). The BR is a task of rotating two iron balls in the palm counterclockwise, which has been used in many studies (Nojima et al., 2012; Suzuki et al., 2013; Horiba et al., 2019). We explained the BR task to the participants, showed them how it worked, and made them actually perform the task in order to understand it. However, we avoided participants learning the BR task and only aimed to get them to understand the content of the task in a short period of time. The protocol of the entire experiment is shown in Figure 1. First, cerebral hemodynamics during the BR-MI task was measured. A block design was used for the measurements, in line with previous research (Wriessnegger et al., 2008; Amemiya et al., 2010; Iso et al., 2016; Matsuo et al., 2020). The participants were instructed to perform the BR-MI task for 30 s and then maintain a resting condition for 40 s. NIRS measurement takes about 5 min depending on the block design. We instructed the participants to perform MI while feeling the muscles as if they were actually performing the motions. We also instructed them to maintain the same posture as that taken during the MI task while they rested and relaxed without thinking of anything. They were instructed to execute the MI task with their eyes closed during MI, as well as during rest, and not to move. In addition, after we fully explained the experimental protocol to the participants, the flow of the task was guided with beeping sounds during the experiment. The measurement of cerebral hemodynamics was performed a total of three times: before and after the initial training of the BR task and after the final training.

Thus, to determine the relationship between cerebral hemodynamics and task competence, participants completed five 1-min sessions of BR tasks involving real movements as the initial training, and the number of BR task completions was measured at each session. The rest period between sessions was about 3 min. Further, participants were asked to self-practice as the final training for improving task competence before completing the sixth 1-min session of BR tasks. The physical training (final) was performed until the subject was satisfied, and the number of BR task completions was measured at the sixth session. The final training was designed for participants to reach the level of competence sufficient to carry out BR tasks. They were asked to self-practice at their own pace, avoiding effects from muscle or mental fatigue. About an hour was given for self-practice. Furthermore, subjective vividness of MI was evaluated three times after NIRS measurement using the VAS (Lotze and Halsband, 2006; Ikeda et al., 2012). The subjects marked a location on a 100-mm horizontal line, the two ends of which were labeled “0 = None at all” and “100 = Very vivid image,” according to the vividness of the imagery they experienced.

### NIRS Measurement and Analysis

**FIGURE 2:**
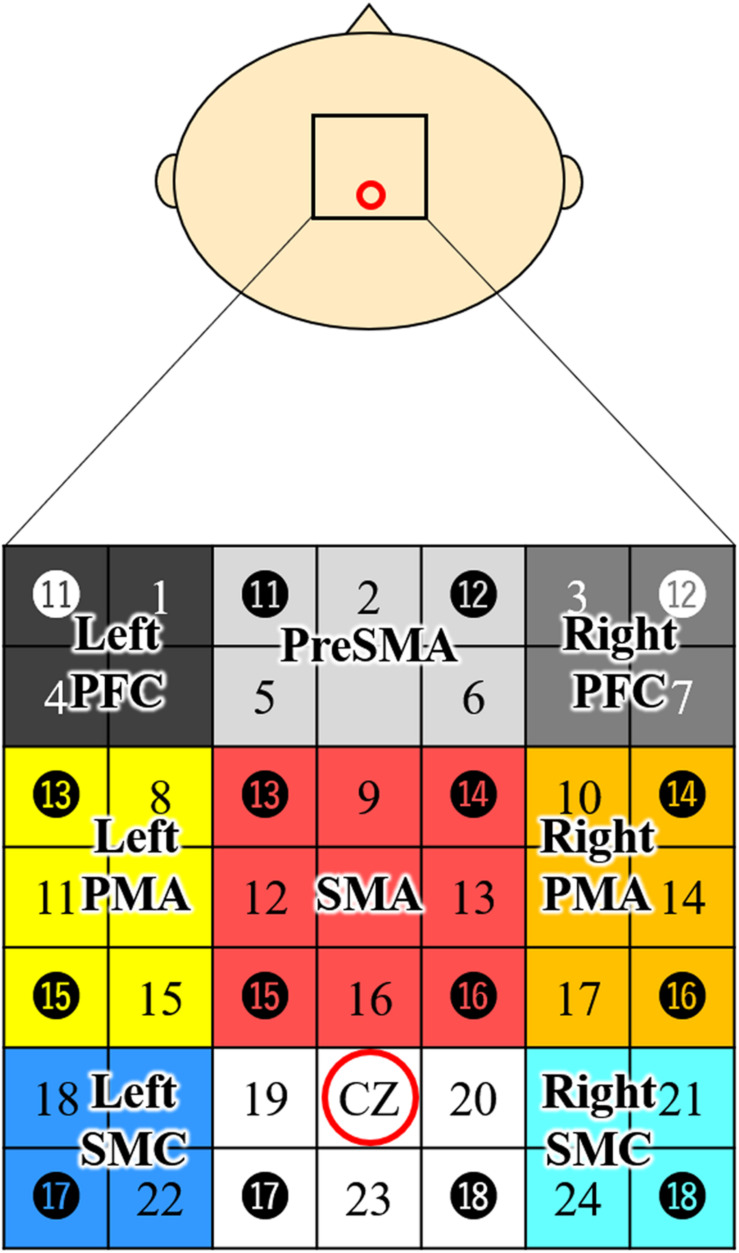
Channel configuration of the 4 × 4 optode probe set. The 24-channel NIRS probe set was positioned over the motor areas. Black circles indicate the positions of NIRS emitters and detectors. The black numbers represent the channels, and the colored boxes show the regions of interest. According to the international 10–20 placement system, Cz was used as a marker position to ensure replicable placement of the optodes. Left PFC, left prefrontal cortex; Right PFC, right prefrontal cortex; Pre-SMA, pre-supplementary motor area; SMA, supplementary motor area; Left PMA, left pre-motor area; Right PMA, right pre-motor area; Left SMC, left somatosensory motor cortex; Right SMC, right somatosensory motor cortex; NIRS, near-infrared spectroscopy.

For NIRS measurements, we used a 24-channel system (ETG-4000; Hitachi Medical Co., Tokyo, Japan) equipped with 4 × 4 optode probe sets (eight incident lights and eight detector fibers), resulting in a total of 24 channels at an inter-optode distance of 3.0 cm. The NIRS channels were placed according to the international 10–20 system, and the Cz position was used as a marker to ensure replicable placement of the optodes (Okamoto et al., 2004; Tsuzuki et al., 2007). A total of eight regions of interest (ROIs) were selected based on previous studies (Hatakenaka et al., 2007; Amemiya et al., 2010; Sagari et al., 2015). The optodes were positioned using a custom-made cap that covered the right and left dorsolateral prefrontal cortex (PFC), pre-SMA, SMA, dorsal PMA, and somatosensory motor cortex (SMC). The areas and optodes covering them were as follows: left SMC, channels 18 and 22; right SMC, channels 21 and 24; SMA, channels 9, 12, 13, and 16; pre-SMA, channels 2, 5, and 6; left PMA, channels 8, 11, and 15; right PMA, channels, 10, 14, and 17; left PFC, channels 1 and 4; and right PFC, channels 3 and 7 (Figure 2). Channels 19, 20, and 23 were not further analyzed.

**FIGURE 3:**
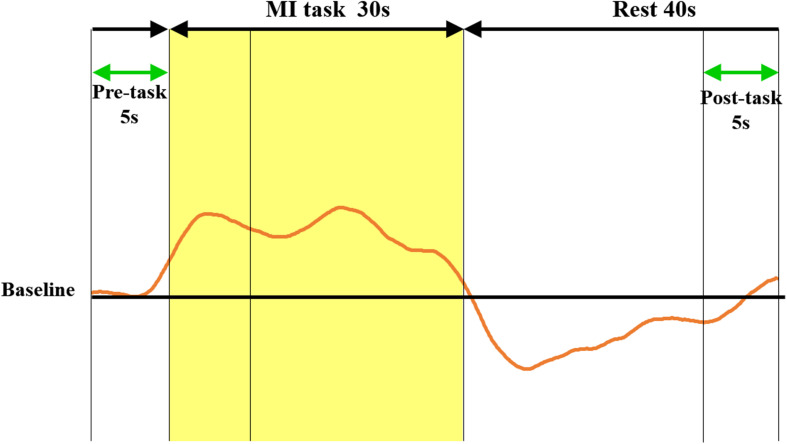
Methods of calculating oxy-Hb change and Z score. The red curve represents the NIRS waveform in the sham case. This waveform was created by averaging the data measured over three cycles in a block design. Linear fitting was applied to the data between the pre-task (5 s) and post-task (5 s) periods (green arrows). The shaded yellow frame indicates the task period, and the non-shaded frame shows the rest period. The vertical axis represents oxy-Hb concentration (mMmm), and the horizontal axis represents the time course. Z-scores of oxy-Hb measured between 0 and 5 s during the pre-task were calculated. The mean value score measured between 5 and 30 s during the task was calculated. oxy-Hb, oxygenated hemoglobin; NIRS, near-infrared spectroscopy.

The continuous-wave NIRS system uses two different wavelengths (625 and 830 nm), which were both used in this study. Relative changes in the absorption of near-infrared light were sampled at 10 Hz, and these values were converted to changes in the concentration of oxy-Hb and deoxygenated hemoglobin (deoxy-Hb) based on the modified Beer-Lambert approach (Cope and Delpy, 1988; Obrig and Villringer, 2003). We used changes in the oxy-Hb concentration as an indicator of fluctuations in the regional cerebral blood volume, as an earlier NIRS signal study using a perfused rat brain model proposed that oxy-Hb and not deoxygenated hemoglobin, is the most sensitive parameter for an activation study (Hoshi et al., 2001). Oxy-Hb is an indicator of local neural activity, rather than of fluctuations in the regional cerebral blood volume (Hoshi et al., 2001). We determined the pre-task baseline as the mean over the 5 s prior to the task period, and the post-task baseline as the mean over the last 5 s of the post-task period (Figure 3). We applied linear fitting to the data between these two baselines (Marumo et al., 2009; Pu et al., 2012). The data were converted into Z-scores using values at 0–5 s from the onset of resting as baselines. We used the average measured 5–30 s after the task had started, considering the time required for changes in oxy-Hb. The average was calculated for each of the eight ROIs. Channels with high noise levels were marked using a 3.0 Hz high-pass filter, and noise components were separated and analyzed using wave analysis. Channels whose standard deviation exceeded 0.08 were assumed to be influenced by excessive noise and were thus excluded (Iso et al., 2016; Matsuo et al., 2020). In addition, during NIRS measurement, the contraction of participants’ thenar muscles was monitored using electromyography (MYOTRACE400 EM-501, Sakai Medico, Japan). Electromyography is a technique that provides biofeedback of muscle activity. During NIRS measurements, motion was monitored by electromyography, which was set to sound when an EMG of 50 μV or higher appeared. Subjects with clear muscle activity during NIRS measurements were excluded, but there were no subjects excluded in this study. For statistical analyses, one-way repeated measures analysis of variance (ANOVA) was performed for the number of BR task completions and the VAS score, followed by a Bonferroni *post hoc* test. To measure oxy-Hb changes, two-way ANOVA was performed, with training and ROI as factors, followed by the Bonferroni *post hoc* test. Five hundred pieces of bootstrap oxy-Hb data were generated for each ROI and VAS in before, initial, and final trainings by randomly drawing a series of actual sample data from the oxy-Hb and VAS score to elicit the difference of variable data between before, initial, and final trainings due to the limited actual sample size. This bootstrap resampling method is widely used in demographic studies (Suzuki et al., 2020). Then, differences in the bootstrap oxy-Hb data between eight ROIs and three training phases (i.e., before, initial, and final trainings) were compared by two-way ANOVA. In addition, a generalized linear model with a gaussian distribution was used to estimate the relationship between the bootstrapping oxy-Hb and VAS scores (Kawanabe et al., 2018). The significance level was set at 5%. The statistical software used was IBM SPSS statistics 26 for one-way ANOVA and two-way ANOVA, Python language for bootstrapping, and R 3.5.2 software (R Foundation for Statistical Computing, Vienna, Wien, Austria) for generalized linear model analysis.

## Results

The number of BRs in each session, VAS scores before and after the initial training, and after the final training (pre, post, and post 2), and the oxy-Hb in each brain area are shown in Figures 4–13. Figure 4 shows the time course of oxy-Hb and deoxy-Hb in each ROI (Z-score). The results of the generalized linear model analysis for predictors of VAS scores are shown in Table 1.

**FIGURE 4:**
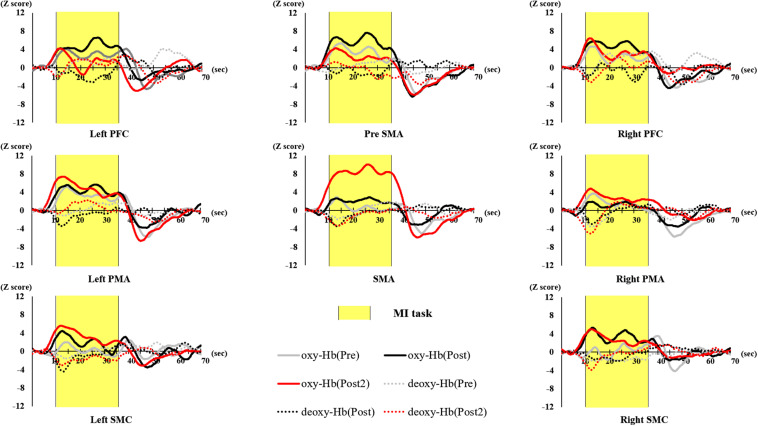
Time course of hemodynamic signal changes for pre- and post- training (initial and final) in each ROI. The vertical axis represents Z score of oxy-Hb and deoxy-Hb, and the horizontal axis represents the time course. The blue line represents deoxy-Hb through all training. The figure shows changes in oxy-Hb and deoxy-Hb during the task in the Left PFC, Right PFC, Pre-SMA, Left PMA, Right PMA, SMA, and Left SMC. Shaded yellow frame indicates the task period. Left PFC, left prefrontal cortex; Right PFC, right prefrontal cortex; pre-SMA, pre-supplementary motor area; SMA, supplementary motor area; Left PMA, left pre-motor area; Right PMA, right pre-motor area; Left SMC, left somatosensory motor cortex; Right SMC, right somatosensory motor cortex; oxy-Hb, oxygenated hemoglobin; deoxy-Hb, deoxygenated hemoglobin; ROI, region of interest.

**FIGURE 5:**
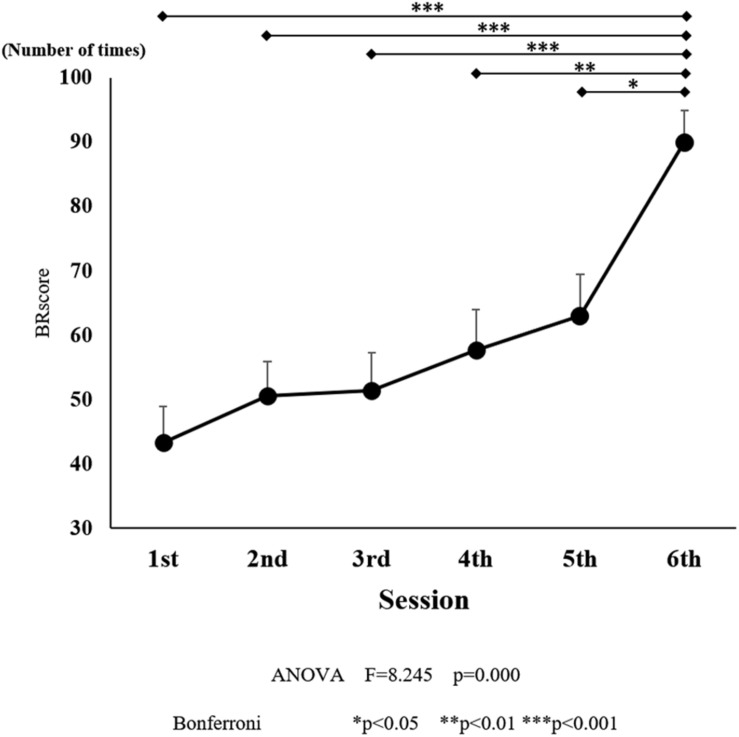
Ball rotation score for each session. The line connecting the dots indicates that there was a significant difference in the *post hoc* analysis. Vertical bars represent the standard error. ^∗^*P* < 0.05, ^∗∗^*P* < 0.01, and ^∗∗∗^*P* < 0.001.

**FIGURE 6:**
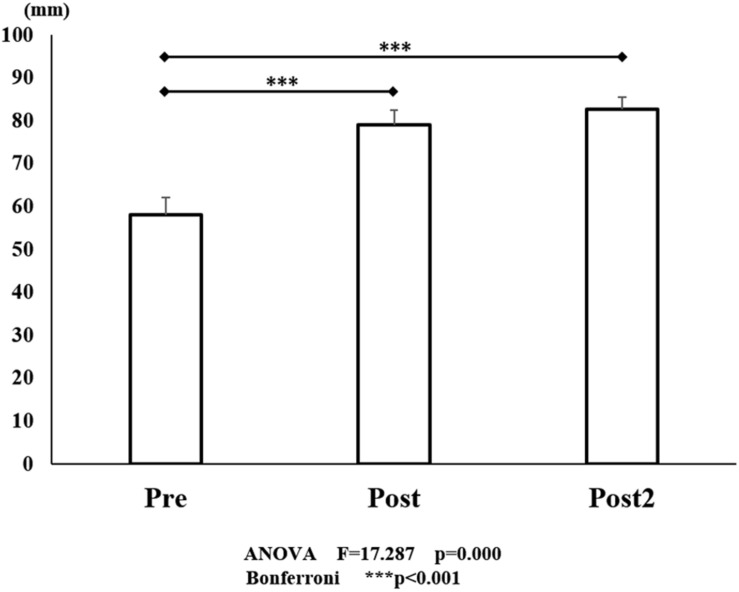
VAS scores for pre- and post- training (initial and final). The line connecting the dots indicates that there was a significant difference on performing identified in the *post hoc* analysis. Vertical bars represent the standard error. ^∗^*P* < 0.05, ^∗∗^*P* < 0.01, and ^∗∗∗^*P* < 0.001. VAS, visual analog scale.

**FIGURE 7:**
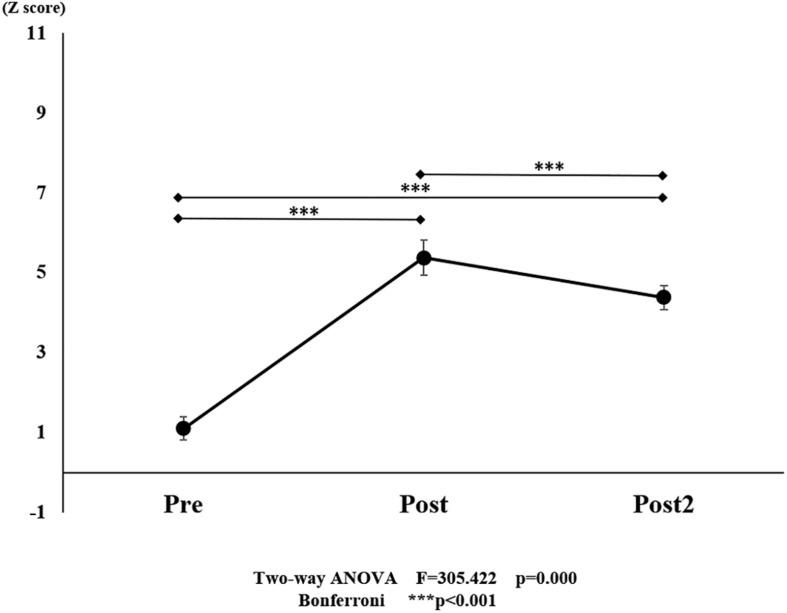
Changes in oxy-Hb pre- and post-training (initial and final) for all ROI. The line connecting the dots indicates that there was a significant difference in the *post hoc* analysis. Vertical bars represent the standard error. ^∗^*P* < 0.05, ^∗∗^*P* < 0.01, and ^∗∗∗^*P* < 0.001. oxy-Hb, oxygenated hemoglobin; ROI, region of interest.

**FIGURE 8:**
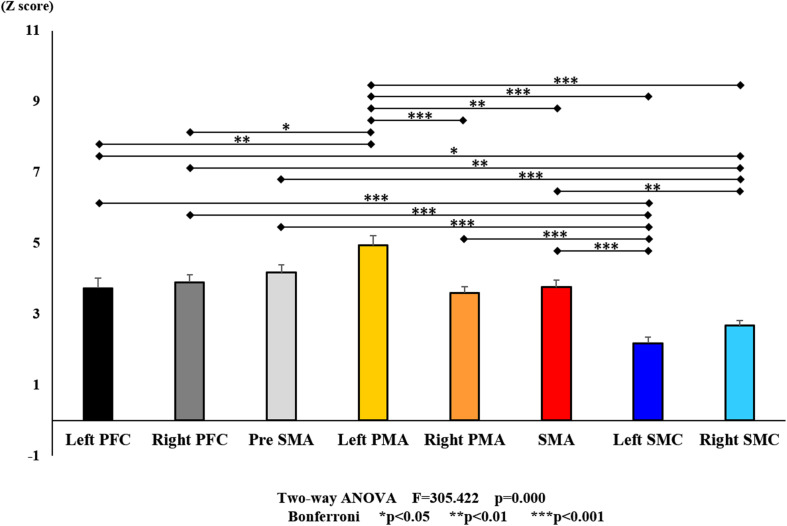
Changes in all stages of oxy-Hb pre- and post-training (initial and final) for each ROI. The line connecting the dots indicates that there was a significant difference in the *post hoc* analysis. Vertical bars represent the standard error. Left PFC, left prefrontal cortex; Right PFC, right prefrontal cortex; pre-SMA, pre-supplementary motor area; SMA, supplementary motor area; Left PMA, left pre-motor area; Right PMA, right pre-motor area; Left SMC, left somatosensory motor cortex; Right SMC, right somatosensory motor cortex; oxy-Hb, oxygenated hemoglobin; ROI, region of interest.

**FIGURE 9:**
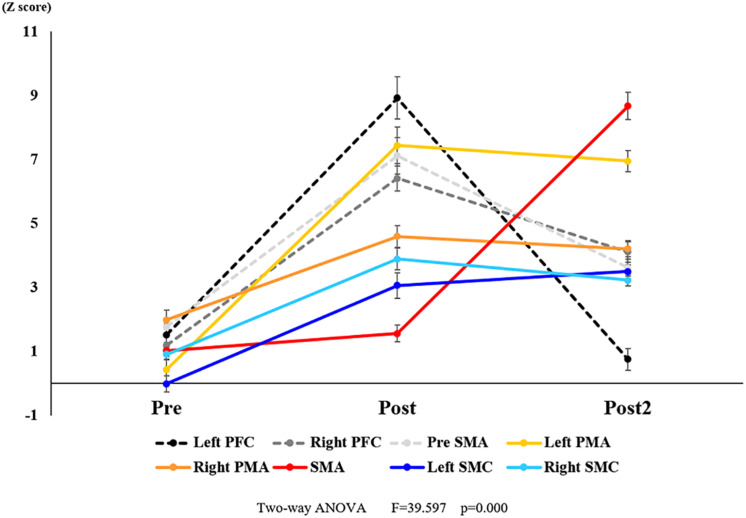
Changes in oxy-Hb for each ROI pre- and post-training (initial and final). The Z score of oxy-Hb changes showed a significant interaction. Black dotted line represents the Left PFC. Dark gray dotted line represents the Right PFC. Light gray dotted line represents the pre-SMA. Yellow line represents the Left PMA. Orange line represents the Right PMA. Red line represents the SMA. Blue line represents the Left SMC. Light blue line represents the Right SMC. Vertical bars represent the standard error. Left PFC, left prefrontal cortex; Right PFC, right prefrontal cortex; pre-SMA, pre-supplementary motor area; SMA, supplementary motor area; Left PMA, left pre-motor area; Right PMA, right pre-motor area; Left SMC, left somatosensory motor cortex; Right SMC, right somatosensory motor cortex; oxy-Hb, oxygenated hemoglobin; ROI, region of interest.

**FIGURE 10:**
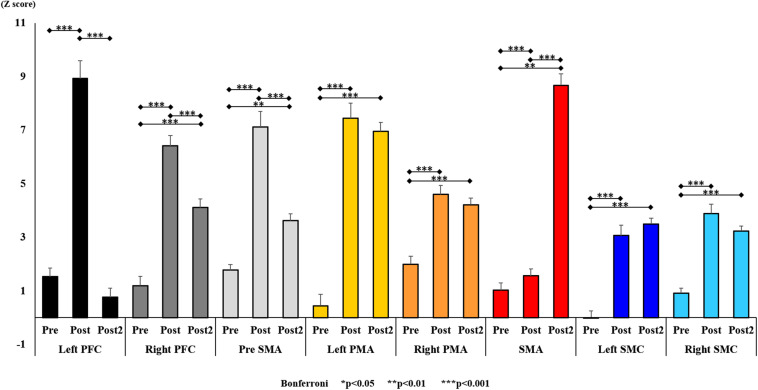
Changes in oxy-Hb for each ROI pre- and post-training (initial and final). The line connecting the dots indicates that there was a significant difference identified in the *post hoc* analysis. Vertical bars represent the standard error. ^∗^*P* < 0.05, ^∗∗^*P* < 0.01, and ^∗∗∗^*P* < 0.001. Left PFC, left prefrontal cortex; Right PFC, right prefrontal cortex; pre-SMA, pre-supplementary motor area; SMA, supplementary motor area; Left PMA, left pre-motor area; Right PMA, right pre-motor area; Left SMC, left somatosensory motor cortex; Right SMC, right somatosensory motor cortex; oxy-Hb, oxygenated hemoglobin; ROI, region of interest.

**FIGURE 11:**
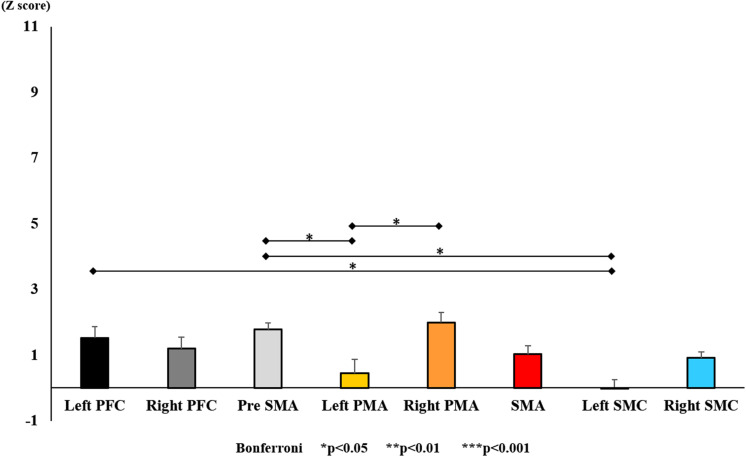
Changes in oxy-Hb for each ROI pre-training (initial round). The line connecting the dots indicates that there was a significant difference in the *post hoc* analysis. Vertical bars represent the standard error. ^∗^*P* < 0.05, ^∗∗^*P* < 0.01, and ^∗∗∗^*P* < 0.001. Left PFC, left prefrontal cortex; Right PFC, right prefrontal cortex; pre-SMA, pre-supplementary motor area; SMA, supplementary motor area; Left PMA, left pre-motor area; Right PMA, right pre-motor area; Left SMC, left somatosensory motor cortex; Right SMC, right somatosensory motor cortex; oxy-Hb, oxygenated hemoglobin; VAS, visual analog scale; ROI, region of interest.

**FIGURE 12:**
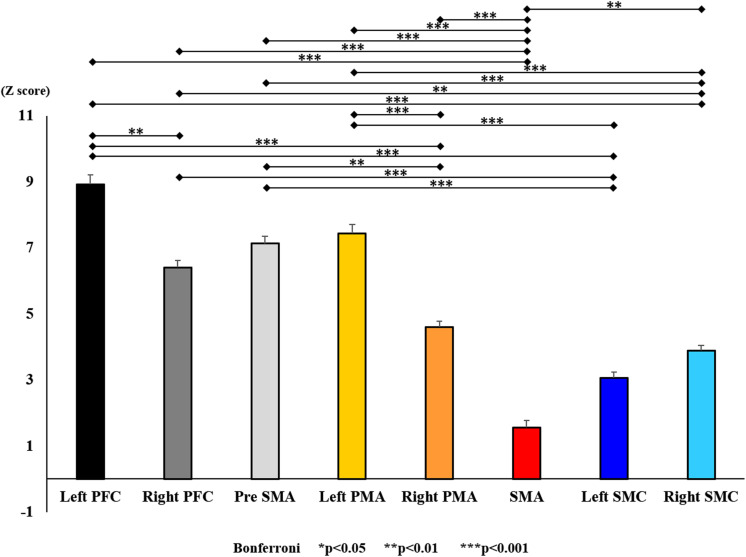
Changes in oxy-Hb for each ROI post-training (initial round). The line connecting the dots indicates that there was a significant difference in the *post hoc* analysis. Vertical bars represent the standard error. ^∗^*P* < 0.05, ^∗∗^*P* < 0.01, and ^∗∗∗^*P* < 0.001. Left PFC, left prefrontal cortex; Right PFC, right prefrontal cortex; pre-SMA, pre-supplementary motor area; SMA, supplementary motor area; Left PMA, left pre-motor area; Right PMA, right pre-motor area; Left SMC, left somatosensory motor cortex; Right SMC, right somatosensory motor cortex; oxy-Hb, oxygenated hemoglobin; ROI, region of interest.

**FIGURE 13:**
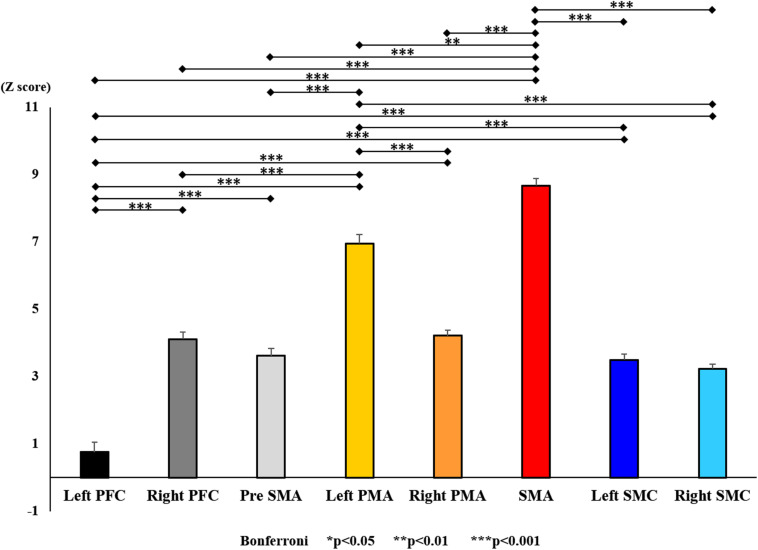
Changes in oxy-Hb for each ROI post-training (final). The line connecting the dots indicates that there was a significant difference in the *post hoc* analysis. Vertical bars represent the standard error. ^∗^*P* < 0.05, ^∗∗^*P* < 0.01, and ^∗∗∗^*P* < 0.001. Left PFC, left prefrontal cortex; Right PFC, right prefrontal cortex; pre-SMA, pre-supplementary motor area; SMA, supplementary motor area; Left PMA, left pre-motor area; Right PMA, right pre-motor area; Left SMC, left somatosensory motor cortex; Right SMC, right somatosensory motor cortex; oxy-Hb, oxygenated hemoglobin; ROI, region of interest.

**TABLE 1:** The results of the generalized linear model analysis for predictors of VAS score.

ROI	Coefficient	Standard error	*t* value	*P* value	
Left PFC	0.0374	0.037	1.021	0.307	
Right PFC	0.1454	0.05	2.908	0.001	**
Pre-SMA	0.1866	0.046	4.069	0	***
Left PMA	0.2129	0.038	5.553	0	***
Right PMA	0.1649	0.06	2.77	0.001	**
SMA	0.3654	0.05	7.321	0	***
Left SMC	0.3398	0.059	5.763	0	***
Right SMC	0.2203	0.073	3.025	0.001	**

The coefficients across seven ROIs ranged between 0.15 and 0.36. *P < 0.05, **P < 0.01, and ***P < 0.001. Left PFC, left prefrontal cortex; Right PFC, right prefrontal cortex; pre-SMA, pre-supplementary motor area; SMA, supplementary motor area; Left PMA, left pre-motor area; Right PMA, right pre-motor area; Left SMC, left somatosensory motor cortex; Right SMC, right somatosensory motor cortex; ROI, region of interest; VAS, visual analog scale.

There was a significant effect of training on the number of BRs [*F*_(5, 90)_ = 8.245, *P* < 0.001; Figure 5]. The number of BRs increased with training, and there was a significant difference observed between the sessions six (*P* < 0.05) compared to those in the initial training session. After the final training session, the average number of BRs was 86.1, which was larger than that observed in the five initial training sessions.

A significant effect on the VAS score was also seen, with the VAS score improved by training [*F*_(2, 45)_ = 17.287, *P* < 0.001; Figure 6]. There was a significant improvement from 40.1 mm (before the initial training) to 65.0 mm (*P* < 0.001) (after the initial training). However, although the score improved to 73.3 mm after the final training (*P* < 0.001), there was no significant difference between the VAS score after the initial training and that after the final training (*P* = 1.000).

With regard to oxy-Hb levels, a main effect of training [*F*_(2, 11976)_ = 305.422, *P* < 0.001] was noted, with significant oxy-Hb changes observed in the pre- and post-initial and post final training (Figure 7). The oxy-Hb changes were highest after the post initial training and slightly decreased after the post final training. Further, a main effect of ROI was noted [*F*_(7, 11976)_ = 16.798, *P* < 0.001], indicating that oxy-Hb changes did vary between different regions (Figure 8). *Post hoc* tests also showed a significant difference between ROIs, indicating large oxy-Hb changes in the left PMA. There was an interaction between training and ROI [*F*_(14, 11976)_ = 39.597, *P* < 0.001], and oxy-Hb levels in the eight ROIs changed in differing directions pre- and post-training (Figure 9). *Post hoc* tests showed a significant increase in oxy-Hb changes in the post initial training, and maintenance or a decrease oxy-Hb changes in post final training (Figure 10). In the SMA, oxy-Hb changes increased with each training. There was no significant difference in oxy-Hb changes in many ROIs in the pre initial training, and the overall oxy-Hb changes were not observed (Figure 11). In the pre-initial training, the pre-SMA showed significant oxy-Hb changes when compared to the left SMC and Left PMA (*P* < 0.05), the right SMA showed significant oxy-Hb changes when compared to the left PMA (*P* < 0.05), and the left PFC showed significant oxy-Hb changes when compared to the left SMC (*P* < 0.05) (Figure 11). In the post initial training, increase of oxy-Hb changes were observed in the overall ROIs. The left PFC showed oxy-Hb changes, and significant differences were observed compared to the right PFC (*P* < 0.01), right PMA, SMA, and bilateral SMC (*P* < 0.001) (Figure 12). Additionally, left PMA and pre-SMA showed significant oxy-Hb changes compared to right PMA (*P* < 0.01), SMA, and bilateral SMC (*P* < 0.001) (Figure 12); compared with other regions, the SMA had significantly lower oxy-Hb changes, which was significantly different from all, except for left SMC (*P* < 0.01, *P* < 0.001) (Figure 12). In the post final training, the SMA showed highest oxy-Hb changes which was significantly different from all other ROIs (*P* < 0.01, *P* < 0.001), and followed by the left PMA (Figure 13). The left PFC showed significantly lower oxy-Hb changes than all other regions (*P* < 0.01, *P* < 0.001) (Figure 13).

The generalized linear model indicated that the oxy-Hb changes for seven ROIs were significant predictors of VAS score. The coefficients across seven ROIs ranged between 0.15 and 0.36 (Table 1).

## Discussion

### Changes in the Number of BRs After Training

The number of BRs increased in every successive session, i.e., compared to the first session, it significantly increased in the second session and so on. Despite short-term training, the number increased significantly; thus, we consider that there was task learning to a certain extent. Compared to the previous study by Kawashima et al. (1998), the participants were not considered to have reached the level of complete learning based on the number of sessions and performance and reached the equivalent degree of learning after the final training. In the final training, instead of repeating the session, BR tasks were performed over time until the subjects were satisfied. Since a significant difference was found between Session 6 and the other sessions after the final training, it is considered that Session 6 was the stage where the tasks were learned considerably.

### Changes in Subjective MI Vividness Before and After Training

There was a significant improvement in the VAS score after training, and the MI clarity improved. A previous study has shown that MI clarity improves with learning of tasks (Robin et al., 2007; Lawrence et al., 2013). The present study also showed learning of the task by training, which is considered to have affected the improvement in subjective MI clarity. However, since there was no significant difference between after the initial training and after the final training, compared to the degree of learning, the changes in the MI clarity are not considered to be completely related proportionally. At a stage where a certain degree of learning is obtained, the stage of the MI clarity is considered high; thus, in the case of a high degree of learning, further analysis may be necessary. As MI vividness improved noticeably through the initial training, no significant difference was observed in the final training. Thus, although there was a positive relationship between MI vividness and task competence, the subjective vividness of MI was relatively high from the early round of training due to exposure to tasks.

### Changes in the Oxy-Hb Levels Before and After Training

The main effect of training was noted, and significant oxy-Hb changes were observed after the initial and final training, compared to before the initial training. The results of this study matched those of previous studies in which oxy-Hb changes in the motor-related areas increased as the MI vividness increased (Mihara et al., 2012; Kober et al., 2014, 2018). The training facilitated learning of the MI task and enabled participants to form MI more vividly, increasing the oxy-Hb levels.

However, in this study, the oxy-Hb changes decreased in the motor-related area after the final training in which the learning of MI tasks was promoted. The difference between this and previous research is that this research was conducted by adopting the tasks used for motor learning (Mihara et al., 2012; Kober et al., 2014, 2018). Previous studies have stated that brain activation is high when the task is difficult, and not activated enough when the task is low in difficulty (Hatakenaka et al., 2007; Amemiya et al., 2010; Sagari et al., 2015). Considering that the tasks were being learned after the final training, it is considered that the MI performed after the final training was relatively easy to carry out. Therefore, it is considered that the difficulty level of the task is at the stage where subjects performed MI easily, and the hemodynamic signal changes during MI are also considered to have decreased as an overall change in the motor-related area.

However, it has also been reported that hemodynamic signal changes during MI differ depending on the ROI (Wriessnegger et al., 2014; Iso et al., 2016; Matsuo et al., 2020), and this study also confirmed the main effect of ROI. The hemodynamic signal changes in the left PMA showed significantly high activation. The PMA has been reported to be deeply involved in motor learning, and activation of the PMA was consistent with previous studies (Tyszka et al., 1994; Hikosaka et al., 1996; Nakamura et al., 1998; Sakai et al., 1998; Kasess et al., 2008; Guillot et al., 2012). In addition, other ROIs showed higher activity compared to the bilateral SMC. The PFC and pre-SMA are also areas involved in early learning of exercise (Nakamura et al., 1998; Sagari et al., 2015), and SMA has been reported to activate during MI (Amemiya et al., 2010; Iso et al., 2016). Similarly, in this study, significantly higher activity was observed in the bilateral PFC, bilateral PMA, pre-SMA, and SMA. In addition, the bilateral SMC showed low activation in this study. Previous imaging studies have shown that SMC activity directly relates to movement output (Obrig et al., 1996; Christensen et al., 2000), while other studies showed increased activation of the primary motor cortex during MI with TMS (Lotze et al., 1999). Furthermore, some studies using NIRS during MI have shown activation of the primary motor cortex. Although the SMC is activated during MI, its role is in movement output, and it is possible that for this reason the hemodynamic signal changes were lower than for other ROIs.

However, in addition to the main effects of training and ROI, interactions were observed, and it was clarified that the hemodynamic signal changes differ depending on the ROI. In particular, the SMA showed an increase in the hemodynamic signal changes as training progressed compared to other ROIs. The SMA is activated in the late stage, suggesting its activation when habituation of movements, including motor planning and preparation, has already been established (Tyszka et al., 1994; Hikosaka et al., 1996; Nakamura et al., 1998; Sakai et al., 1998; Kasess et al., 2008; Guillot et al., 2012). It is an area responsible for performing MI-related activity, including motor preparation (Mihara and Miyai, 2016). With progression of learning the MI task, participants were able to evoke MI more vividly, leading to a gradual increase in changes in cerebral blood flow in the SMA, which was one of the ROIs in this study. However, the SMA did not show high activation until the learning stage was quite advanced. The results of this study were consistent with those of previous studies regarding motor learning (Tyszka et al., 1994; Hikosaka et al., 1996; Nakamura et al., 1998; Sakai et al., 1998; Kasess et al., 2008; Guillot et al., 2012).

Therefore, our results show that training increased the hemodynamic signal changes in all ROIs, with the greatest increase observed in the PMA. However, only SMA the showed an increase in the hemodynamic signal with training compared to other ROIs.

### Relationship Between Oxy-Hb Changes and Subjective Vividness of MI

A generalized linear model showed that oxy-Hb changes in seven ROIs, other than the left PFC, were significant predictors of VAS score. In particular, the SMA coefficient was the highest, which was a significant predictor for estimating VAS scores, and was shown to be most associated with VAS. Since the change in VAS and the change in oxy-Hb of the SMA due to training are also similar, the oxy-Hb change in the SMA best represents the vividness of MI. The SMA was reported to be activated in previous NIRS studies during MI (Mihara et al., 2012; Kober et al., 2014, 2018; Matsuo et al., 2020). Our present results showed that the selection of these areas was adequate for examining the MI vividness in terms of changes in cerebral hemodynamics using NIRS as an objective index for these changes. Our results were also consistent with the findings of a previous study (Mihara and Miyai, 2016), which reported that these areas are responsible for MI. An important aspect in mental practice is how vividly an individual can perform MI; if MI vividness can be evaluated neurophysiologically, MI can be used effectively in rehabilitation. MI is already used in neurofeedback systems such as Brain Computer Interface (BCI) and Functional electrical stimulation therapy (Khan et al., 2015; Koo et al., 2016; Likitlersuang et al., 2018). Our results demonstrate that hemodynamic signal changes in the SMA are associated with MI vividness, which can be used not only for mental practice but also for other techniques such as BCI.

Therefore, the hemodynamic signal changes in motor related areas centered on the SMA using NIRS might be used as a neurophysiological assessment for MI vividness.

### Limitations

In the present study, we used BR as the learning task, which primarily relies on fingers dexterity; therefore, the relationship between MI vividness and cerebral hemodynamic changes obtained in this study might be limited to MI of the upper extremities.

In addition, this study was conducted on healthy subjects; when using MI vividness as an evaluation tool in patients with stroke it is necessary to consider the injured area.

### Conclusion

The BR task used in the present study showed a clear motion learning from practicing the actual motions, thereby causing significant improvement in subjective MI vividness. In terms of hemodynamic signal changes, a main effect of training and ROIs were observed.

Moreover, the coefficients for estimating relationships are highest in the SMA, a key predictor for estimating VAS scores, which suggests that it is the best region for detecting MI vividness. Thus, our findings suggest that future studies should examine neurophysiological indices for MI vividness based on the hemodynamic signal changes of SMA.

## Data Availability Statement

The raw data supporting the conclusions of this article will be made available by the authors, without undue reservation.

## Ethics Statement

The studies involving human participants were reviewed and approved by the Ethics Committee of the Medical Corporation Toujinkai. The patients/participants provided their written informed consent to participate in this study.

## Author Contributions

NI, TM, KF, and THi conceived and designed the experiments. NI, TM, KF, WM, THa, FI, and THi performed the experiments. NI, MM, KF, WM, and THi analyzed the data. TM, THa, KC, MS, and THi created the experimental program. NI, TM, FI, and THi wrote the article. All authors discussed the results and contributed to the final manuscript.

## Conflict of Interest

The authors declare that the research was conducted in the absence of any commercial or financial relationships that could be construed as a potential conflict of interest.

## Abbreviations

ANOVA: analysis of variance
BCI: brain computer interface
BR: ball rotation
deoxy-Hb: deoxygenated hemoglobin
fMRI: functional magnetic resonance imaging
KVIQ: Kinesthetic and Visual Imagery Questionnaire
MI: motor imagery
NIRS: near-infrared spectroscopy
oxy-Hb: oxygenated hemoglobin
PET: positron emission tomography
Left PFC: left prefrontal cortex
Right PFC: right prefrontal cortex
pre-SMA: pre-supplementary motor area
SMA: supplementary motor area
Left PMA: left pre-motor area
Right PMA: right pre-motor area
Left SMC: left somatosensory motor cortex
Right SMC: right somatosensory motor cortex
ROI: region of interest
SMA: supplementary motor area
SMC: somatosensory motor cortex
TMS: transcranial magnetic stimulation
VAS: visual analog scale.
